# Energy saving strategies of honeybees in dipping nectar

**DOI:** 10.1038/srep15002

**Published:** 2015-10-08

**Authors:** Jianing Wu, Heng Yang, Shaoze Yan

**Affiliations:** 1 Division of Intelligent and Biomechanical Systems, State Key Laboratory of Tribology, Department of Mechanical Engineering, Room 3407, Building 9003, Tsinghua University, 100084, Beijing, PR China; 2 Department of Automotive Engineering, Tsinghua University, Beijing, 100084, PR China

## Abstract

The honeybee’s drinking process has generally been simplified because of its high speed and small scale. In this study, we clearly observed the drinking cycle of the Italian honeybee using a specially designed high-speed camera system. We analysed the pattern of glossal hair erection and the movement kinematics of the protracting tongue (glossa). Results showed that the honeybee used two special protraction strategies to save energy. First, the glossal hairs remain adpressed until the end of the protraction, which indicates that the hydraulic resistance is reduced to less than 1/3 of that in the case if the hairs remain erect. Second, the glossa protracts with a specific velocity profile and we quantitatively demonstrated that this moving strategy helps reduce the total energy needed for protraction compared with the typical form of protraction with constant acceleration and deceleration. These findings suggest effective methods to optimise the control policies employed by next-generation microfluidic pumps.

Natural drinking strategies have elicited considerable interests from multiple disciplines^1^. The drinking cycles of animals such as cats^2^, dogs^3^, bats^4^ and hummingbirds^5^,^6^ have been observed and analysed explicitly in varying degrees. However, the drinking processes of some insects, such as butterflies, mosquitoes, and honeybees, cannot be easily observed because of their relatively small mouthparts and/or high intake rates. Special structures found in the mouthparts of these insects add to the complexity of their drinking strategies, which are worthy of extensive research^7^. The Italian honeybee (*Apis mellifera ligustica*) is a typical honeybee and its mouthparts have been widely studied. This insect forms a sucking tube with its galeae and labial palpi while drinking nectar, and its tongue (glossa) produces a dipping motion based on forward protraction and backward retraction to load and unload the fluid during drinking (Fig. 1)^8^. Recently, Kim *et al.*^9^ examined a bumblebee drinking nectar and established a quantitative viscous dipping model that explained the natural preference for a nectar concentration of 35% in flowers pollinated by bees. Later, Yang *et al.*^10^ investigated the microstructures of glossal hairs and modified the viscous dipping model by adding the effects of erectable glossal hairs. These two models can explain, to some extent, the drinking strategies of honeybees and their natural evolution choice of optimal nectar concentration. However, these models have been simplified because they cannot observe the delicate drinking behaviour of the bees’ mouthparts at microlevel, thereby neglecting some important and interesting strategies.

In the present study, we employed a well-designed high-speed viewing system to clearly observe the drinking process of the Italian honeybee, particularly the erection pattern of the glossal hairs and the protraction kinematics of the dipping glossa. We demonstrated that the honeybees can significantly reduce the total energy needed for protraction by using the two specific mechanisms above.

## Results

### Erection pattern of the glossal hairs

**Figure 1:**
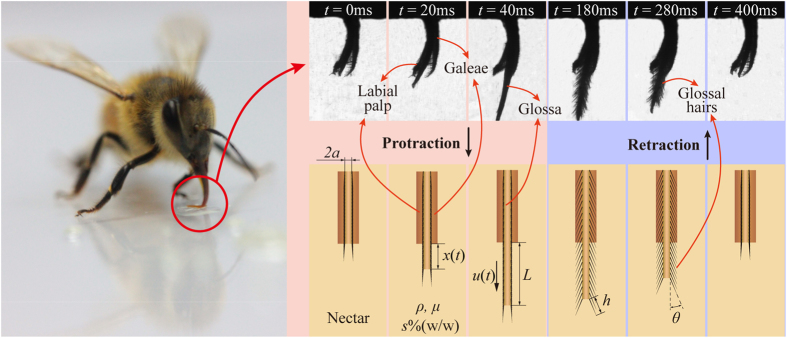
Illustration of the bee’s drinking cycle. (**a**) A honeybee feeding nectar under the camera (*Apis mellifera ligustica*), captured by Jianing Wu. (**b**) Selected frames showing the honeybee’s drinking cycle. The first three photographs show the protraction process where the glossa elongates to dip into the nectar and the glossal hairs are adpressed. The last three photographs show the retraction process where the glossa erects its hairs to trap nectar and shortens to load the nectar into its mouthparts. The white text above the photographs indicates the timings of these frames. (**c**) Physical model illustrating the drinking process. The glossa is simplified into a cylinder with dense hairs and the labial palpi and galeae are treated as a sucking tube that surrounds the cylinder. The cylinder has a diameter 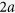, total length 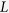, movement velocity 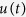 and length 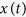 while protracting the nectar; the hair has a height 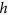 and an erection angle *θ*; and the nectar has a density *ρ*, mass concentration 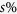 and viscosity *μ*.

We analyzed the frame-by-frame high-speed photographs of the honeybee during drinking and selected six typical drinking events. The first three photographs in Fig. 1(b) show the glossa extending into the nectar, and the last three photographs illustrate the withdrawal of the glossa to unload nectar into the sucking tube. The total drinking cycle lasted for approximately 400 ms, which corresponded to the typical dipping frequency of 2.5 Hz^9^. The protraction and retraction times were 50 and 350 ms, respectively, indicating that the honeybee protracted much faster than it retracts. The erection pattern of the glossal hairs is an important feature of the drinking process. The glossal hairs were not erected until the beginning of the retraction process, suggesting that the honeybee kept the hairs adpressed when protracting its glossa into the nectar. This behaviour was positively controlled by the honeybee because the glossal hairs should have been passively erected by hydrodynamic resistance. During retraction, the hairs were still not fully erected, with an erection angle of 0° < *θ* < 90°. This characteristic is probably ascribed to the attempt of the honeybee to optimize the erection angle by balancing the two effects caused by the increased erection angle, namely, the enhanced fluid drag and the trapping of more nectar during one cycle.

### Movement kinematics of the protracting glossa

**Figure 2:**
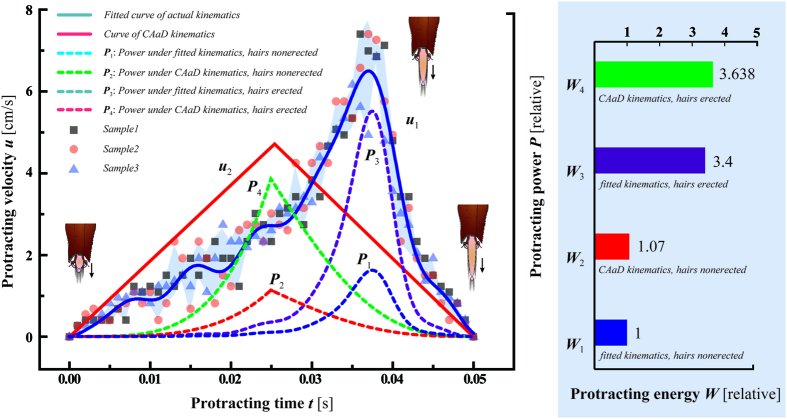
The honeybee’s glossal protraction kinematics saves energy. (**a**) The scatter plot shows three independent protraction velocities measured using high-speed video and the light blue area indicates the error band of the velocities. The bold blue curve represents the Fourier kinematics 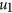, which fits the scatter plot well. The bold red curve shows the constant-acceleration-and-deceleration (CAaD) kinematics 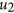. The dashed blue line 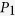 and the dashed red line 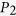 indicate the protraction power levels under the fitted kinematics and CAaD kinematics with the glossal hairs adpressed, respectively. The dashed purple line 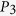 and the dashed green line 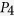 indicate the protraction power levels under the fitted kinematics and CAaD kinematics with the glossal hairs erected, respectively. The insets show the bee proboscis to illustrate the three corresponding steps during the glossal protraction process. (**b**) Comparisons of total protracting energy under different cases are presented. The energy needed under fitted kinematics with glossal hairs adpressed, 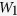 is normalized as 1.

We measured the vertical length of the part of the glossa that was immersed in the nectar over time *x*(*t*) during protraction (the galeae and labial palpi surrounded the part of the glossa; thus, only the glossal tip was immersed in the nectar). Additionally, we derived the velocity profile, *u*(*t*), by difference. We measured three independent protraction events of the same honeybee specimen and averaged the velocities during these protractions (Supplementary Table SI). The scatter plot in Fig. 2(a) shows that the honeybee exhibited steady protraction kinematics. We used the curve-fitting toolbox in Matlab (R2013b, Math Works, Natick, MA, USA) to derive an analytical expression for the continuous function, that is, *u*(*t*), and found that a seven-order Fourier function can fit the curve well (*R*^2^ = 0.9853):


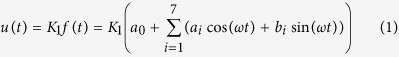


where 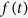 is the Fourier function; The parameters *a*_0_, *ω*, *a*_*i*_, and *b*_*i*_ (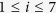) were calculated by Matlab to obtain the optimal fit for the scatter plot. The coefficient 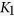 (137 μm/cm) converts the sizes in the high-speed photographs into those for an actual honeybee. The bold blue line in Fig. 2(a) presents the fitted curve.

## Discussion

As shown in Fig. 1(c), we simplify the glossa as a cylinder, and the labial palpi and galeae as a sucking tube. To extend the glossa into the nectar, the honeybee must overcome the viscous resistance and inertial force of the tongue. The power required for viscous drag can be estimated as *P*_*v*_ ∼ *μLu*^2^, where *μ* is the viscosity of the nectar and *L* is the protraction length of the tongue, and the power needed for tongue acceleration can be estimated as 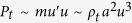, where *a* is the radius of the glossa and *ρ*_*t*_ is the density of the glossa. Since the ratio 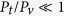, the effect of 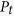 can be neglected^9^. The viscous drag can be written as 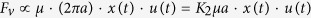, where *K*_2_ is a proportionality coefficient. By combining equation (1) and 
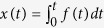
, the power needed to overcome viscous drag is:


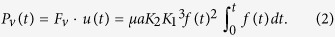


We can determine the benefits of keeping the glossal hairs adpressed during protraction from equation (2). If the honeybee erects the hairs, the glossa will be covered by them and the cylinder’s radius will increase from *a* to (*a* + *h* cos*θ*), which will lead to a significant increase in 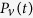. We obtained measurements using scanning electron microscope (SEM) images of *a* ≈ 50 μm and *h* ≈ 170 μm, and since *θ* ≈ 45°, we can calculate that (*a* + *h* cos*θ*)/*a* = 3.4, which means that erecting the hairs will increase the resistance by more than three times. Therefore, the honeybee has evolved an energy-saving glossal hair erection pattern, where the hairs adhere to the glossa to reduce viscous drag during protraction, whereas the hairs erect to trap more nectar in a single cycle during retraction^10^.

We also considered the specific protraction kinematics of the honeybee’s glossa. As shown in Fig. 2(a), the total protraction time was 50 ms, where the glossa’s movement velocity increased slowly initially before decreasing sharply. We found that the total protraction length of the glossa *L* was 1 mm, the total time required for one drinking cycle was 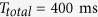 (Fig. 1(a)) and the average movement velocity during one cycle was 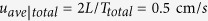. However, Fig. 2(a) shows that the maximum velocity of the glossa during protraction, 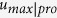, could be as high as 7 cm/s and the average protraction velocity 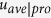 was 2.3 cm/s, both of which are much higher than 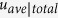. We also examined the retraction process carefully and found that the glossa moved slowly at a uniform speed of 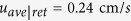, indicating that the honeybee moves its glossa much faster during protraction than retraction. This phenomenon suggests that the honeybee worker consumes most of its energy during protraction in the overall drinking process. We compared the specific protraction kinematics and their effects on the energy consumption of the honeybee with the constant-acceleration-and-deceleration (CAaD) kinematics. The CAaD kinematics exhibited a common protraction pattern, where the glossa velocity increased linearly for half of the protraction period, before decreasing linearly during the next half of the protraction period (bold red line 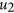 in Fig. 2(a)). According to equation (1), we calculated and plotted the protraction power with these two different kinematics using Matlab (dashed blue line 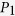 and dashed red line 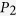 in Fig. 2(a)). This plot showed that the maximum pumping power of the kinematics fitted for a honeybee was greater than that of the CAaD kinematics. The CAaD kinematics also had a lower working power during the latter part of the protraction. When we calculated the total energy required for the overall protraction process by integrating the two protraction power curves, however, we found that the energy requirement under the fitted kinematics was 7% less than that under the CAaD kinematics. This reduction was reliable because we performed similar calculations for three directly measured velocity profiles and also determined reductions in the total protraction energy (see Supplementary Information). The energy-saving strategy of a honeybee may be explained as follows. First, the fitted kinematics may have considerably reduced the pumping power during the first half of the protraction process because the velocity initially increased very gradually. Second, although the pumping power increased sharply from the middle of the protraction process in the fitted kinematics, the honeybee overcame this disadvantage by reducing the velocity much more sharply than that in the CAaD kinematics. This behaviour narrowed the time range when a high level of power should be generated.

Additionally, we list the energy consumptions under four scenarios, determined by whether the honeybee erects her hairs or not during protraction, and whether it takes the fitted or CAaD kinematics, in Fig. 2(b) to show the honeybee’s two drinking strategies. First, keeping the hairs close to the glossa during protraction can reduce the resistance to less than 1/3 of that when the hairs are erect. Second, the total energy required under the fitted Fourier protraction kinematics is 7% less than that under CAaD kinematics.

The microstructures in various organisms, articularly insects, have demonstrated interesting functions that may offer promising applications^11^. Hairy structures in biological systems are found to significantly influence friction and adhesion control^12^,^13^,^14^, interlocking mechanisms^15^, water transport^16^, self-cleaning systems^17^ and water absorption of plants^18^. In the current work, we investigated another feature of the insects’ hairs in which the moving pattern of glossal hairs plays an essential role in the bee’s nectar feeding. We used a video capture system to elucidate the drinking process of the Italian honeybee. We examined the erection pattern of the glossal hairs and the protraction kinematics of the dipping glossa. We demonstrated that the honeybee achieves an efficient energy-saving mechanism by adopting the two drinking strategies. Cally *et al.*^19^ found that the bat tongue is like a specialized mop, that is, the erection pattern of the hair-like papillae in the tongue of the bat helps nectar trapping. The tongue of a honeybee is similar to a specialized brush controlling the erection pattern of glossal hairs. Both bat and honeybee need to extend their tongues deep into their liquid food sources. Thus, the trapping strategy in nectar feeding through a hairy system is deployed across species and a natural optimization process with respect to the living environment of these species. Therefore, examining the anatomy of the glossa of a honeybee is an interesting topic for future research. Moreover, the mechanism by which a honeybee coordinates different structures within the tongue to control hair erection should be explored. The drinking strategy of a honeybee may inspire some new concepts to facilitate the design of micropumps^20^.

## Methods

**Figure 3:**
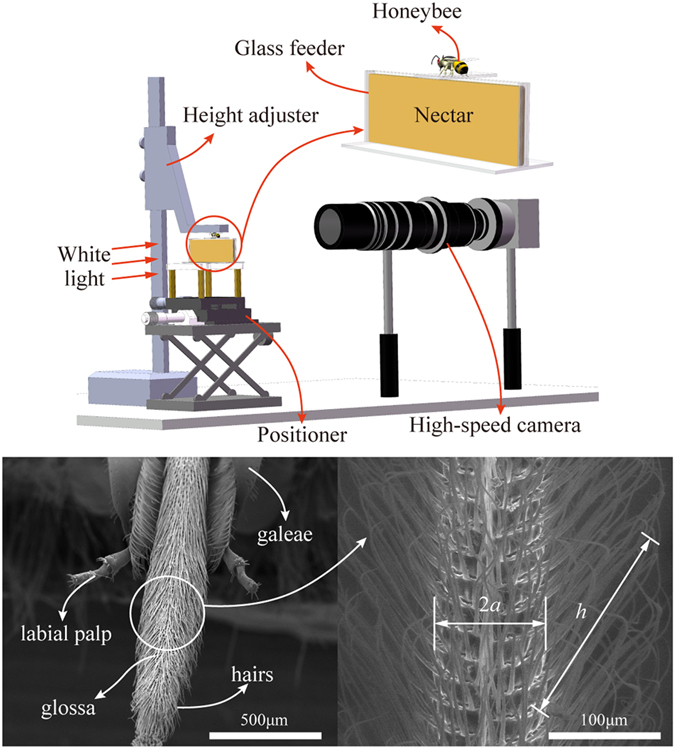
Experimental setup and SEM images. (**a**) Experimental setup. The system comprised a positioner, height adjuster, light source, glass feeder containing nectar and a high-speed camera with a microscope. This figure is drawn by Heng Yang. (**b**) The mouthparts consist of a pair of labial palpi, a pair of galeae and a glossa covered by dense hairs. (**c**) The enlarged part of a typical glossa, by which the diameter of the glossa, 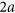, and the height of the hair, 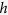, are measured.

Honeybee specimens were collected from Tsinghua University, Beijing, China (40.000153 °N, 116.326414 °E) and housed in a wooden beehive at constant temperature of 25 °C and 50% humidity. No specific permissions were required for these locations/activities. We confirm that the field studies did not involve endangered or protected species. The experimental equipment comprised a positioner, height adjuster, glass feeder containing artificial nectar (sucrose solution), light source and a high-speed camera (Olympus, iSpeed TR, Japan) with a microscope (Keyence, VH-Z50L, Japan) (Fig. 3a). During the experiment, the honeybee was glued to the height adjuster via its thorax and it was moved up and down with the adjuster, thereby allowing its mouthparts to reach the level of the artificial nectar. We filmed a honeybee drinking a 35% (wt./wt.) sucrose solution from a lateral shooting angle at a frequency of 1000 frames per second (see Supplementary Movie S1). Experiments were repeated with 10 honeybees, which all showed similar drinking behaviour. In total, ten mouthpart specimens were dehydrated for experiment within 4 hours after they were captured in the inspection box. They were first processed by *Pentylene Glycol* and washed by phosphate buffer, then dehydrated using graded ethanol from 50% to 100%, and finally desiccated for 10 minutes using a drying box. These ten specimens were observed using SEM to obtain detailed information about the mouthparts’ structure and morphology (Fig. 3(b,c)). Also, the sizes of the mouthparts were directly measured in SEM figures.

## Additional Information

**How to cite this article**: Wu, J. *et al.* Energy saving strategies of honeybees in dipping nectar. *Sci. Rep.* **5**, 15002; doi: 10.1038/srep15002 (2015).

## Supplementary Material

Supplementary Information

Supplementary Video
