# Supplementary material for: Energy saving strategies of honeybees in dipping nectar
Source: Sci Rep. 2015 Oct 8;5:15002. doi: 10.1038/srep15002 (PMC4597227; doi:10.1038/srep15002)
Supplement: Supplementary Information [file srep15002-s1.doc]

Supplementary Information for

**Energy saving strategies of honeybees in dipping nectar**

Jianing Wu, Heng Yang and Shaoze Yan*

*Corresponding author. E-mail: [yansz@mail.tsinghua.edu.cn](mailto:yansz@mail.tsinghua.edu.cn)

# Observation of the honeybee’s drinking process

We film the honeybee drinking the 35% (wt/wt) sucrose solution from a lateral shooting angle, at a frequency of 1000 frames per second. The video showing the process is Movie S1.

# Derivation of the glossa’s protracting velocities

The high-speed photography gives us a series of tiff images representing the mouthpart’s behaviors per 1ms. We directly measure from the images the displacements of the glossa and then divide these displacements by 1ms to get the velocities. Since the microscope has enlarged the bee’s mouthpart, we use the coefficient $K_{1}$, which is around $137\text{μm/cm}$, to convert the velocities derived before to the the velocities in real world. Three independent drinking cycles are measured and the data are shown in table SI.

Table SI. Data of protracting velocities measured from high speed photos and then converted to real sizes.

| $t/\text{ms}$ | $\begin{matrix} u_{1}\left(t\right) \\ /[\mathrm{cm}/\mathrm{s}] \end{matrix}$ | $\begin{matrix} u_{2}\left(t\right) \\ /[\mathrm{cm}/\mathrm{s}] \end{matrix}$ | $\begin{matrix} u_{3}\left(t\right) \\ /[\mathrm{cm}/\mathrm{s}] \end{matrix}$ | $t/\text{ms}$ | $\begin{matrix} u_{1}\left(t\right) \\ /[\mathrm{cm}/\mathrm{s}] \end{matrix}$ | $\begin{matrix} u_{2}\left(t\right) \\ /[\mathrm{cm}/\mathrm{s}] \end{matrix}$ | $\begin{matrix} u_{3}\left(t\right) \\ /[\mathrm{cm}/\mathrm{s}] \end{matrix}$ |
| --- | --- | --- | --- | --- | --- | --- | --- |
| 0 | 0 | 0 | 0 | 26 | 3.014 | 2.74 | 3.151 |
| 1 | 0.274 | 0.274 | 0.137 | 27 | 3.151 | 2.603 | 3.014 |
| 2 | 0.411 | 0.548 | 0.274 | 27 | 3.425 | 4.247 | 3.151 |
| 3 | 0.411 | 0.685 | 0.411 | 29 | 2.74 | 3.151 | 3.425 |
| 4 | 0.411 | 0.411 | 0.548 | 30 | 3.836 | 4.247 | 3.288 |
| 5 | 0.411 | 0.411 | 0.822 | 31 | 3.425 | 4.658 | 4.384 |
| 6 | 0.685 | 0.685 | 0.959 | 32 | 4.658 | 4.247 | 4.658 |
| 7 | 0.411 | 0.822 | 0.959 | 33 | 5.069 | 5.754 | 5.069 |
| 8 | 1.233 | 1.096 | 1.096 | 34 | 4.932 | 5.754 | 5.343 |
| 9 | 1.096 | 0.822 | 1.096 | 35 | 5.343 | 5.343 | 6.165 |
| 10 | 1.233 | 1.096 | 0.822 | 36 | 7.398 | 6.576 | 5.617 |
| 11 | 1.096 | 0.411 | 1.233 | 37 | 6.987 | 7.398 | 4.932 |
| 12 | 1.507 | 0.822 | 1.233 | 38 | 6.85 | 7.261 | 6.85 |
| 13 | 1.233 | 2.329 | 1.37 | 39 | 7.124 | 5.754 | 4.795 |
| 14 | 1.507 | 1.918 | 1.507 | 40 | 4.795 | 4.932 | 3.562 |
| 15 | 1.918 | 0.685 | 1.233 | 41 | 3.425 | 2.329 | 3.014 |
| 16 | 1.507 | 2.329 | 1.918 | 42 | 3.425 | 3.151 | 2.74 |
| 17 | 1.918 | 1.918 | 1.096 | 43 | 2.329 | 1.918 | 2.055 |
| 18 | 2.329 | 0.822 | 1.918 | 44 | 1.918 | 1.507 | 1.37 |
| 19 | 1.918 | 1.507 | 2.74 | 45 | 1.507 | 1.507 | 1.918 |
| 20 | 1.918 | 1.233 | 2.329 | 46 | 1.233 | 0.822 | 1.096 |
| 21 | 1.233 | 2.603 | 2.329 | 47 | 1.096 | 0.411 | 0.959 |
| 22 | 2.329 | 2.74 | 2.192 | 48 | 0.822 | 0.822 | 0.822 |
| 23 | 3.014 | 2.74 | 2.466 | 49 | 0.411 | 0.274 | 0.411 |
| 24 | 2.74 | 2.329 | 2.329 | 50 | 0 | 0 | 0 |
| 25 | 2.329 | 3.014 | 2.603 | - | - | - | - |

# Curve fitting and the calculation of protracting power

We first average the three columns of velocities in table SI to get the average protracting velocity. Then we use the curve fitting toolbox of Matlab (R2013b, MathWorks, Natick, MA, USA) to fit these data to derive an analytical expression of $u\left(t\right)$. The Fourier function can well fit the data and the result is

| $u\left(t\right)=K_{1}f\left(t\right)=K_{1}\left(a_{0}+\sum_{i=1}^{7} \left(a_{i}\cos\left(\omega t\right)+b_{i}\sin\left(\omega t\right)\right)\right),$ | (1) |
| --- | --- |

where $f\left(t\right)$ is the Fourier function, $a_{0}$, $\omega$ and $a_{i}$, $b_{i}$ ($1\leq i\leq7$) are parameters calculated by Matlab to best fit the data, and $K_{1}$ ($137\text{μm/cm}$) is the coefficient which converts the sizes in high-speed photos to those of the real honeybee. $a_{0}$ and $a_{i}$, $b_{i}$ ($1\leq i\leq7$) are listed in Table SII.

Table SII. Parameters calculated by Matlab to fit the velocity data.

| $a_{0}$ | $a_{1}$ | $a_{2}$ | $a_{3}$ | $a_{4}$ | $a_{5}$ | $a_{6}$ | $a_{7}$ |
| --- | --- | --- | --- | --- | --- | --- | --- |
| 0.1703 | -0.1125 | -0.06924 | 0.02255 | 0.01254 | -0.01682 | -0.008773 | 0.007208 |
| $\omega$ $/[\mathrm{rad}/s]$ | $b_{1}$ | $b_{2}$ | $b_{3}$ | $b_{4}$ | $b_{5}$ | $b_{6}$ | $b_{7}$ |
| 121.2 | -0.1174 | 0.04726 | 0.03128 | -0.01825 | -0.005768 | 0.007946 | 0.007972 |

We have also done similar curve fittings using the data of $u_{1}\left(t\right)$, $u_{2}\left(t\right)$ and $u_{3}\left(t\right)$, respectively and get three continuous functions of $u_{1}\left(t\right)$, $u_{2}\left(t\right)$ and $u_{3}\left(t\right)$ like equation (1).

The protracting power can be written as

| $\begin{matrix} P\left(t\right)=\mu aK_{2}u\left(t\right)^{2}\int_{0}^{t} u\left(t\right)dt \\ =\mu aK_{2}\left(K_{1}f\left(t\right)\right)^{2}\int_{0}^{t} K_{1}f\left(t\right)dt \\ =\mu aK_{2}K_{1}^{3}f\left(t\right)^{2}\int_{0}^{t} f\left(t\right)dt, \end{matrix}$ | (2) |
| --- | --- |

where $\mu$ is the viscosity of the nectar and $K_{2}$ is a proportionality coefficient related to viscous drag. If the glossa protracts at a constant-acceleration-and-deceleration (CAaD) kinematics, the speed would be

| $u_{a}\left(t\right)=\left\{\begin{matrix} \frac{4L}{T_{p}^{2}}t,\left(0<t<T_{p}/2\right) \\ \frac{4L}{T_{p}^{2}}\left(T_{c}-t\right),\left(T_{p}/2<t<T_{p}\right) \end{matrix}\right.,$ | (3) |
| --- | --- |

where $T_{p}$ is the total protraction time 50ms. Working power with respect to time is

| $\begin{matrix} P_{a}\left(t\right)=\mu aK_{2}u_{a}\left(t\right)^{2}\cdot u_{a}\left(t\right)t \\ =\frac{64\mu aK_{2}L^{3}}{T_{p}^{6}}\left\{\begin{matrix} t^{4},\left(0<t<T_{p}/2\right) \\ t\left(T_{c}-t\right)^{3},\left(T_{p}/2<t<T_{p}\right) \end{matrix}\right.. \end{matrix}$ | (4) |
| --- | --- |

We then integrate equation (2) and equation (4) against time in Matlab to gain the total energy needed for protraction and we find that there is a 7% decrease in the total energy needed under the fitted kinematics compared to CAaD kinematics.

In order to test whether 7% is convincible, we done similar calculations using the three independent kinematics $u_{1}\left(t\right)$, $u_{2}\left(t\right)$ and $u_{3}\left(t\right)$, and we find the percentages of reduction in total protracting power are 7.65%, 5.75% and 15.42%, respectively. So it can be proved that the honeybee dose use this special protracting kinematics to save energy.
